# Supplementary material for: Large-scale epidemiology of opisthorchiasis in 21 provinces in Thailand based on diagnosis by fecal egg examination and urine antigen assay and analysis of risk factors for infection
Source: PLoS Negl Trop Dis. 2025 Jul 16;19(7):e0013095. doi: 10.1371/journal.pntd.0013095 (PMC12313068; doi:10.1371/journal.pntd.0013095)
Supplement: S1 Text — (DOCX) [file pntd.0013095.s001.docx]

**Sample size calculation**

The sample size calculation was estimated using a single proportion formula: $n=Z_{\alpha}^{2}\left[ p(1-p) \right]/d^{2}$ where P is the prevalence of *O. viverrini* from previous records in the study sites was 10%, Z is the level of confidence (1.96), and d is 2% marginal error [46]; this gave a minimum sample size of 862 individuals. By adjusting for the long-term study and the attrition rate, the minimum number of participants required in this study was estimated at 1,000 for each site. A total of 21,000 individuals were invited to participate in the project, including those who resided in the study area during the project operation.
