# Supplementary figures and images for: Large-scale epidemiology of opisthorchiasis in 21 provinces in Thailand based on diagnosis by fecal egg examination and urine antigen assay and analysis of risk factors for infection
Source: PLoS Negl Trop Dis. 2025 Jul 16;19(7):e0013095. doi: 10.1371/journal.pntd.0013095 (PMC12313068; doi:10.1371/journal.pntd.0013095)

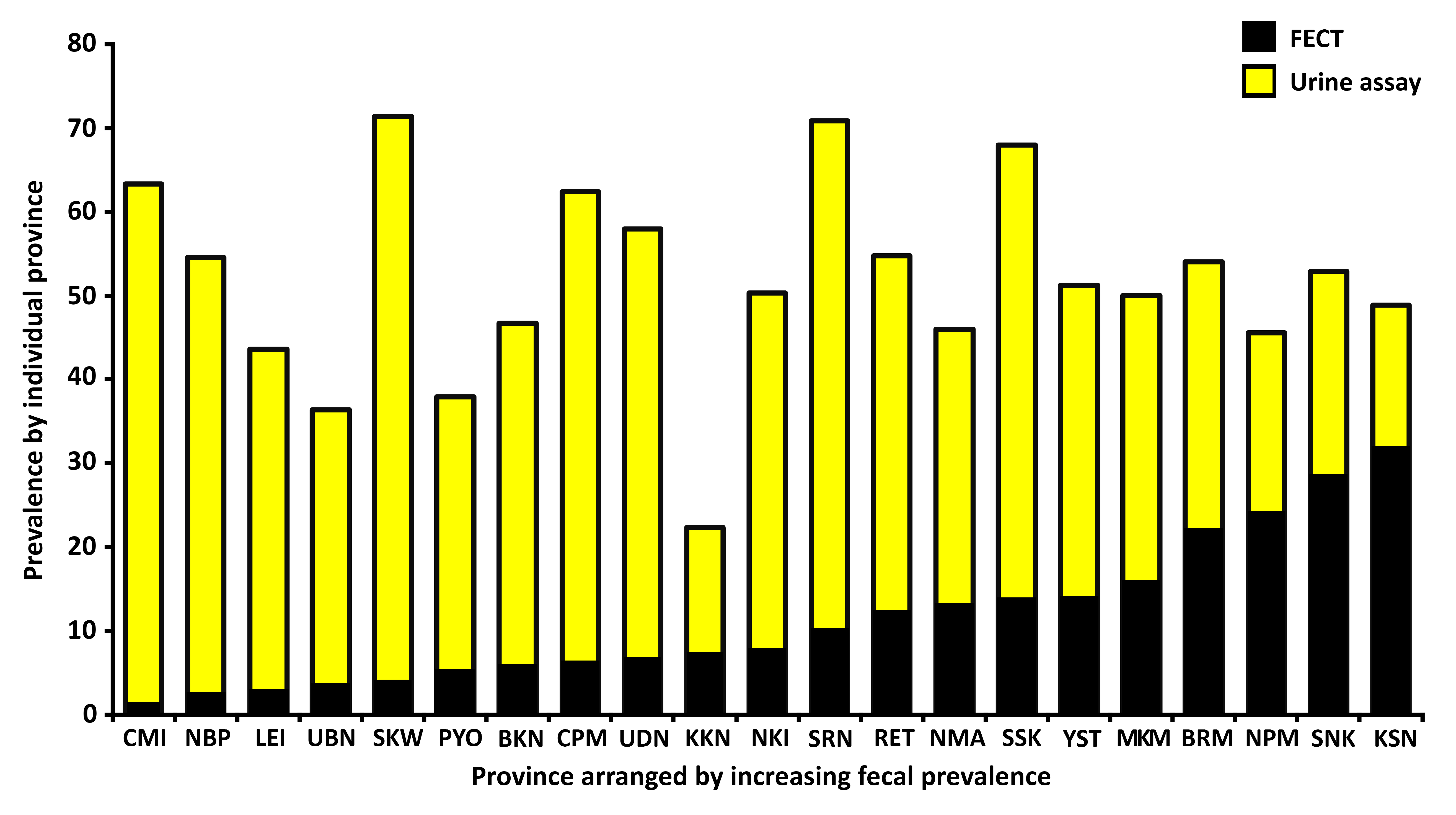

Supplement: S1 Fig — Dark bars indicate prevalence as determined by FECT; superimposed white bars indicate prevalence measured using urinary antigen detection by ELISA. Bars are arranged in order of ascending prevalence based on FECT results. CMI: Chiang Mai, NBP: Nong Bua Lamphu, LEI: Loei, UBN: Ubon Ratchathani, SKW: Sa Kaeow, PYO: Phayao, BKN: Bueng Kan, CPM: Chaiyaphum, UDN: Udon Thani, KKN: Khon Kaen, NKI: Nong Khai, SRN: Surin, RET: Roi Et, NMR: Nakhon Ratchasima, SSK: Si Sa Ket, YST: Yasothon, MKM: Maha Sarakham, BRM: Buriram, NPM: Nakhon Phanom, SNK: Sakon Nakhon, and KSN: Kalasin. (TIF) [file pntd.0013095.s002.tif]

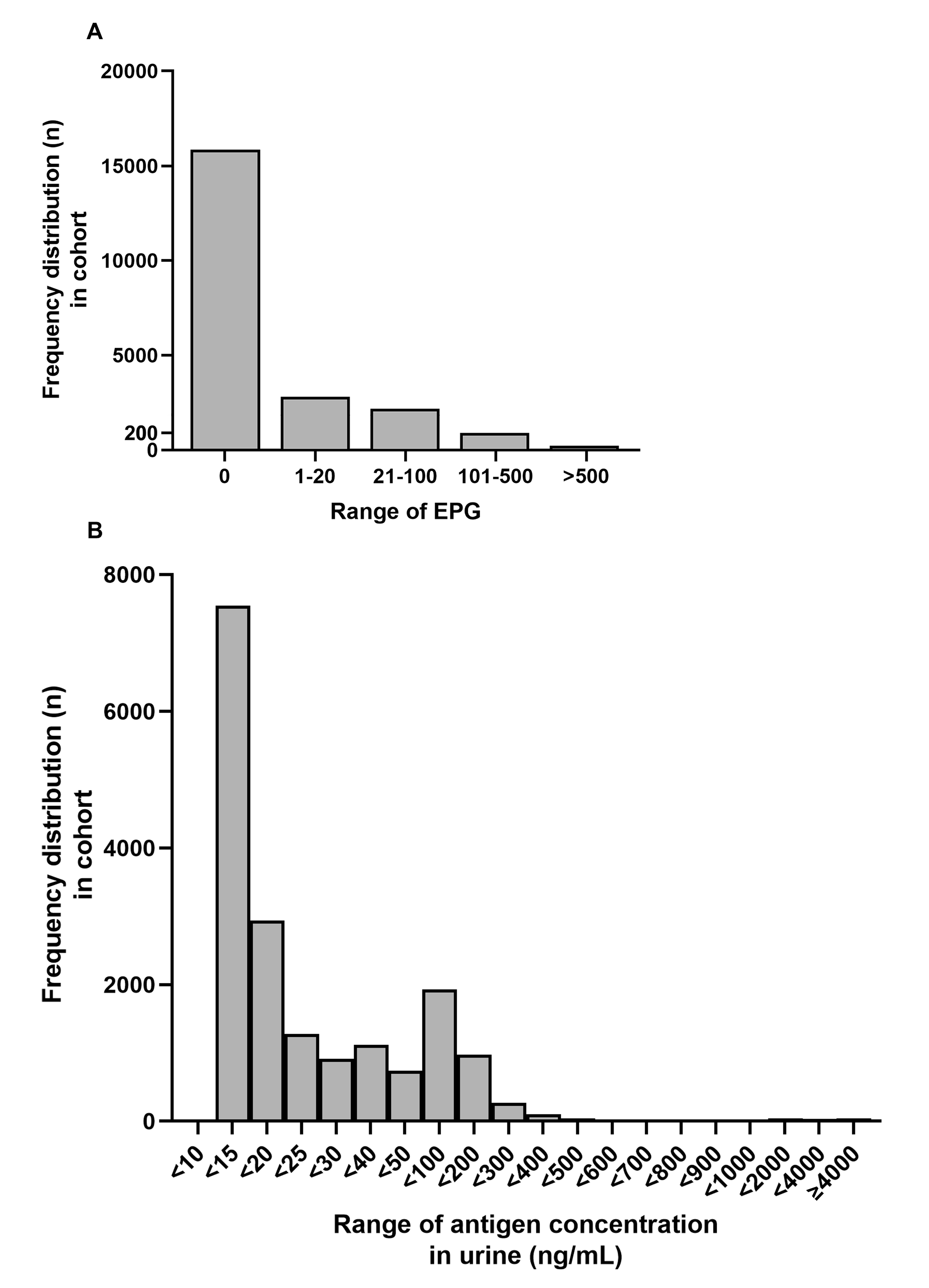

Supplement: S2 Fig — The fecal egg counts frequency (median = 16.9 EPG) (A), and the urine antigen concentration (median = 47.8 ng/ml) among antigen-positive individuals (B) within the sample population in Thailand. (TIF) [file pntd.0013095.s003.tif]
