# Supplementary material for: Large-scale epidemiology of opisthorchiasis in 21 provinces in Thailand based on diagnosis by fecal egg examination and urine antigen assay and analysis of risk factors for infection
Source: PLoS Negl Trop Dis. 2025 Jul 16;19(7):e0013095. doi: 10.1371/journal.pntd.0013095 (PMC12313068; doi:10.1371/journal.pntd.0013095)
Supplement: Attachment — Submitted filename: Reviewer Attachment.docx [file pntd.0013095.s006.docx]

- The author might move the sample size calculation into the supplement section.
- Age groups should be categorized into the same interval, such as 20-129, 30 – 39, and so on. Please consider revising it throughout the manuscript including the analysis and figures.
- From figure 2, why are there more enrolled participants than eligible participants?
- From my perspective, the FECT could directly detect the presence of Ov eggs under the microscope. How can the authors be sure that the positive urine antigen results refer to the presence of Ov eggs in the specimen?
- The FECT has high sensitivity compared to simple smear or the Kato technique. Even compared with PCR, the sensitivity difference is less than fourfold, as presented in this study. Could false positive or cross-reaction play a major role in that difference?
- Before proceeding to compare sensitivity from composite results, the author might consider some critical issues;
  - Those with positive results from FECT and urine antigen and those with negative results from FECT and urine antigen. These are straightforward because we have Ov eggs presented in the specimen.
  - The authors might need to discuss when one test is positive and the other is negative.
  - I doubt the test with negative FECT with positive urine antigen as the authors did not have a reference test such as PCR. The FECT itself is sufficient for being a reference test in community trials. However, if the authors wish to evaluate the sensitivity of urine antigens in this setting, they should compare it with FECT (as a reference test). I strongly not recommend using composite results as a reference test.
  - Please consider this central issue and revise the analysis.
