# Supplementary material for: Large-scale epidemiology of opisthorchiasis in 21 provinces in Thailand based on diagnosis by fecal egg examination and urine antigen assay and analysis of risk factors for infection
Source: PLoS Negl Trop Dis. 2025 Jul 16;19(7):e0013095. doi: 10.1371/journal.pntd.0013095 (PMC12313068; doi:10.1371/journal.pntd.0013095)
Supplement: Attachment — Submitted filename: Reviewer responses_updateA.docx [file pntd.0013095.s008.docx]

**Response to Editor and Reviewer**

**#1 Respond to the Editor (highlighted in yellow)**

- The author might move the sample size calculation into the supplement section.

**Answer:** The sample size calculation has been corrected and moved to the supplement section as S1 sample size calculation file(line 203).

- Age groups should be categorized into the same interval, such as 20-29, 30-39, and so on. Please consider revising it throughout the manuscript, including the analysis and figures.

**Answer:** Following this comment, the age groups in Fig. 3 have been revised. This adjustment has been applied throughout the text, analysis, and figures to maintain uniformity.

- From Figure 2, why are there more enrolled participants than eligible participants?

**Answer:** Thank you for raising this point, we corrected the number of eligible participants as 21,000 individuals and this correction is made in the revised Figure 2.

- From my perspective, the FECT could directly detect the presence of Ov eggs under the microscope. How can the authors be sure that the positive urine antigen results refer to the presence of Ov eggs in the specimen?

**Answer:** Thank you for this point, we add an explanation in the discussion (line 487-493)

Among people tested negative for opisthorchiasis by FECT, this study found that 43.9% were positive by urine ELISA. This finding is consistent with our previous reports (Worasith et al., 2015, 2019, 2022). Conversely, some individuals were fecal egg-positive but urine antigen-negative which also aligned with findings from earlier reports (Worasith et al., 2015, 2019). This discrepancy may result from the misidentification of minute intestinal flukes as O. viverrini or other underlying factors which restrict the passage of eggs into stool, such as biliary pathology require further investigation.

- The FECT has high sensitivity compared to simple smears or the Kato technique. Even compared with PCR, the sensitivity difference is less than fourfold, as presented in this study. Could false positive or cross-reaction play a major role in that difference?

**Answer:** Please see our response above.

- Before proceeding to compare sensitivity from composite results, the author might consider some critical issues:
  - Those with positive results from FECT and urine antigen and those with negative results from FECT and urine antigen. These are straightforward because we have Ov eggs presented in the specimen.

Answer : Please see our response above

- The authors might need to discuss when one test is positive, and the other is negative.

Answer : Please see our response above.

- I doubt the test with negative FECT with positive urine antigen as the authors did not have a reference test such as PCR. The FECT itself is sufficient for being a reference test in community trials. However, if the authors wish to evaluate the sensitivity of urine antigens in this setting, they should compare it with FECT (as a reference test). I strongly not recommend using composite results as a reference test.

**Answer :** In respond to the reviewer suggestion regarding the latent class analysis, we have perform the analysis in R using a multinomial outcome variable, which indicates one of four possible diagnostic states; positive by both FECT and Antigen tests [1,1]; positive for FECT and negative for Antigen [1,0]; positive for the antigen and negative for FECT [0,1]; or negative by both tests [0,0]. The parameters to be estimated by the model are the *O. viverrini* prevalence by province, the sensitivity of FECT, and the sensitivity and specificity of the antigen. If we assumed the specificity of FECT as 0.99. Our results indicate that the sensitivity of FECT is 0.714 with a 95% credible interval (CrI) of 0.649-0.779; the sensitivity of the antigen test is 0.622 (95% CrI 0.603-0.641) and the specificity is 0.512 (95% CrI 0.504-0.519).

Since microscopic examination by FECT cannot differentiate *O.viverrini* and other minute intestinal flukes and other pathological factors which restrict the passage of eggs into stool undermined the reliability of stool examination. While DNA detection by PCR which confirms *O.viverrini* was not done in our study. Whether the analysis by latent analysis will help to increase the accuracy of diagnosis or not is uncertain. We have applied the concept of a primary reference standard using FECT and a composite reference standard which combined multiple methods for diagnosis of opisthorchiasis in multiple endemic communities in Thailand (Worasith et al, 2029, 2929, 2022, Kopoolrat et al, 2022).

The text regarding reference diagnostic method is added to discussion, line 560-566.

Fecal examination is the conventional gold standard for diagnosing opisthorchiasis and other helminth infections. However, an autopsy study demonstrated that worm recovery of Opisthorchis viverrini is more sensitive than fecal egg examination, as the majority of individuals with fewer than 20 worms had no detectable eggs in their feces (Sithithaworn et al., 1991). Therefore, recovering worms from the biliary system is considered a more accurate method for diagnosing opisthorchiasis. However, both FECT and antigen detection indicate the presence of worms in the liver i.e. active opisthorchiasis.

Please consider this central issue and revise the analysis.

**Answer:** Please see our response above.

Diagnostic accuracy in results (beginning from line 421-434) was revised as follows.

Based on matched urine and fecal samples from all study sites, the diagnostic performance of FECT and urine ELISA for opisthorchiasis was assessed based on two reference standards: the primary reference standard (FECT) and a composite reference standard (a combination of FECT and the urine antigen test), as presented in Table 3. Urine ELISA exhibited an overall positive rate of 50.9%, with 49.3% of positive cases occurring among individuals who tested negative by FECT. Conversely, FECT had an overall positive rate of 12.1%, with 38.2% of cases testing negative by urine ELISA. The concordant positive rate for both FECT and urine ELISA was 7.5%.

Using FECT as the primary reference standard, the sensitivity and specificity of urine ELISA were 61.7% and 50.6%, respectively. When the composite reference standard was used, the sensitivity and specificity for urine ELISA were 91.6% and 100%, respectively, while FECT showed sensitivity and specificity of 21.9% and 100%. The observed agreement between the composite reference standard and urine ELISA was almost perfect (kappa = 0.9), whereas the agreement for FECT was slight (kappa = 0.2).

Table 3 is revised as

**Table 3**. **Diagnostic accuracy of urinary antigen detection by enzyme-linked immunosorbent assay (ELISA) and formalin ethyl-acetate concentration technique (FECT) compared with a primary and composite reference standard (n = 18,062).**

a) **FECT as a primary reference standard**

| **Method** | **ELISA result** | **Formalin ethyl acetate concentration technique (FECT)** | | |
| --- | --- | --- | --- | --- |
|  |  | **Positive** | **Negative** | **Total** |
| **ELISA** | **Positive** | 1,359 | 7,830 | 9,187 |
|  | **Negative** | 843 | 8083 | 8,873 |
| **Total** | | 2,202 | 15,860 | 18,062 |

b) **Composite reference standard (combined FECT and urine antigen detection)**

| **Methods** | **ELISA result** | **Composite reference standard** | | |
| --- | --- | --- | --- | --- |
|  |  | **Positive** | **Negative** | **Total** |
| **ELISA** | **Positive** | 9189 | 0 | 9187 |
|  | **Negative** | 843 | 8083 | 8873 |
| **Total** | | 10,032 | 8083 | 18062 |
| **FECT** | **Positive** | 2,202 | 0 | 2,202 |
|  | **Negative** | 7,830 | 8,030 | 15,860 |
| **Total** | | 10,032 | 8,030 | 18,062 |

**Reviewers' Comments:**

**Key Review Criteria Required for Acceptance?**

As you describe the new analyses required for acceptance, please consider the following:

**Methods**
Reviewer #2: I recommend a minor revision to this manuscript, as I do not consider that new investigations, analysis, or experiments are needed in this study.

Regarding the Methods section:

- The objectives are clear and the study design seems appropriate to address the objectives.
- The population is well described and the sample size well justified. Laboratory methods, questionnaire, and statistical analysis are also well described and structured in the Results section.

- Descriptions in the Methods section ensure repeatability/reproducibility of the study, especially the laboratory methods.

**Answer:** Thank you very much for your kind consideration.

I wonder whether the following details could be explained/included:

- Why was the age group younger than 15 years excluded from the study? This could be useful, especially as the recruited cohort of this cross-sectional study is intended to serve as the basis of a longitudinal study (if I understood correctly).

**Answer:** Multiple studies have shown that O. viverrini infection prevalence increases with age due to cumulative exposure from long-term consumption of raw or undercooked fish. Additionally, the risk of hepatobiliary complications, including cholangiocarcinoma, begins to rise after the age of 40 (Khuntikeo et al., 2018). The prevalence of opisthorchiasis is low among children under 15 years of age. To optimize costs, our study focused on individuals over 15 years old who are at higher risk. However, while the prevalence of opisthorchiasis in children is relatively low in Thailand, it can be higher in regions with intense transmission, such as Lao PDR and other endemic areas. Therefore, the risk of infection in children in these high-transmission settings warrants closer attention.

The following texts are added to Material and method section line 203-205

Younger individuals aged <15 years were not recruited as a strategy to focus on groups with a higher likelihood of infection for longitudinal study.

Also in discussion line 571-573.

Another limitation is the exclusion of participants aged < 15 years due to infection risk, leaving a gap in understanding opisthorchiasis transmission in younger age groups.

- Urine samples: If possible, I suggest expressing centrifugation units in RCF (‘g’) instead of rpm, as it is more informative. Line 247 for FECT has both rpm and RCF.

**Answer:** The centrifugation units have been revised as RCF (‘g’) in line 230 and line 239

- Urine samples were frozen (-20 °C) until analysis: Could freezing have an impact on antigen ELISA quantifications? Do similar studies all use frozen urine, or do some of these use freshly collected (or cooled only) urine samples?

**Answer:** Based on our experience, prolonged storage of urine samples (>4 weeks) at freezing temperatures may lead to antigen degradation. To ensure antigen stability, our protocol for urine sample handling involves keeping the samples on ice immediately after collection and during transportation. Upon arrival at the laboratory, the samples are centrifuged to separate the supernatant, then stored at –20 °C. Antigen detection is performed within 2 weeks to maintain antigen integrity. This protocol has been consistently used in our studies with reliable results (Worasith et al., 2015; 2019; 2020).

Fresh urine samples can also be used for antigen detection. After centrifugation or allowing the sample to sediment at room temperature, testing should be completed within 2 hours. If a longer handling time is required, the samples should be kept on ice to preserve antigen stability.

**Results**

Reviewer #1: (No Response)

Reviewer #2: - The structure and presentation of results is overall quite clear, succint, and aligned with what is presented in the Methods section. For example, I find figures Fig 2 and 6 very illustrative and informative while keeping it simple, and I would like to thank the authors for it.
Some details to consider:

- Check consistency in reported overall prevalence based on urine antigen ELISA test (50.3% in Table 1, line 478, line 564; vs. 50.9% in lines 72, 109, 329). Based on numbers of Table 1, 50.3% seems to be correct.

**Answer:** A correction was made in lines 71, 107, and 322

- Line 327: Do the authors maybe prefer to mention the lowest (3.6%) and highest prevalence by FECT?

**Answer:** A correction was made in the result section, line 320.

- Line 330: Highest prevalence by antigen ELISA test is 59.3% in the East region, based on Table 1.

**Answer:** The sentence is revised as

The lowest prevalence was 49.3% in the northern region, and the highest prevalence was 59.3% in the eastern region (Table 1).

- Could the authors elaborate, and possibly include in the Methods or Results section, how the composite reference standard assigned disease status based on FECT and antigen results? Should both diagnostics be positive to classify an individual as positive for opistorchiasis?

**Answer:** Please see the answer to the editor above.

- Line 431: “sensitivity for urine ELISA was 91.1%” vs. Table 3 (91.6%).

**Answer:** Please see revised Table 3.

- Line 444: It should refer to Table 4, not Table 3.

**Answer:** A correction was made.

- Lines 461-462 (educational level) is somewhat redundant with lines 447-449 and could be combined to include aOR data.

**Answer:** As suggested, the sentence is revised in line 451-453 as

People with higher educational backgrounds (secondary school or certificate and higher) had significantly lower odds of infection than those in primary school and lower education (aOR = 0.80, 95 % CI: 0.67-0.95, p=0.013).

- S Fig 1 and S Fig 2 appear to be the same. I believe S Fig 1 is missing.

**Answer:** Thank you. SFig 2 is now added.

- Fig 5: Is Log10 transformation needed, or the best choice for this figure? Would concentration in ng/mL show a more clear increasing trend as intensity of infection (EPG) increases? Or would it be too skewed?

**Answer:** As suggested, Fig. 5 is revised as suggested.

**Conclusions**

Reviewer #1: (No Response)

Reviewer #2: - Conclusions are brief, summarise the results well, and are supported by the data presented.

I would like to suggest the following in the Discussion section:

- Lines 535-538 and 545-547 could be combined to avoid redundancy.

**Answer:** This is done.

- The authors are aware and transparent about the limitations of the study, and this is always appreciated from a reader's perspective. I would maybe add the age group below 15 years old as either a limitation of the study, or an explanation of why this age group was not recruited, if intentional (if the latter, likely in the Methods section instead).

- While brief, the authors do address the public health relevance and next steps (a longitudinal study).

**Answer:** Please see above.

**Editorial and Data Presentation Modifications?**

Use this section for editorial suggestions as well as relatively minor modifications of existing data that would enhance clarity. If the only modifications needed are minor and/or editorial, you may wish to recommend “Minor Revision” or “Accept”.

Reviewer #1: (No Response)

Reviewer #2: - Lines 140-144: I would recommend splitting this long sentence in two or rephrase it to make the reading flow smoother.

**Answer:** The sentence is revised in line 138-142

A national liver fluke control program focused on egg detection and the treatment of infected individuals with the highly effective drug praziquantel from 1987-91. Subsequent nationwide surveys detected declines in the prevalence at a national level, to 11.8% in 1996 and 9.6% in 2001, and in Northeast Thailand, to 12.4% in 1996 and 15.7% in 2001 [12, 13].

- Line 167: Change “we” for “We” after full stop.

**Answer:** A correction was made in line 164.

- Consistency in the use of singular vs plural verbs (was/were) after measurement units: Line 244 “Two grams of fresh stool was processed”, vs line 246, “Three milliliters of ethyl acetate were added”. This might be up to the journal’s guidelines rather than the authors.

**Answer:** A correction was made in line 245.

- Lines 252-254: I believe a verb is missing at the end of this sentence, e.g., “was performed based on…”.

**Answer:** A correction was made as suggested.

- Line 292: The dot is in red colour.

**Answer:** A correction was made in line 292.

- Line 300: “(iii)” is written twice and “(ii)” is missing.

**Answer:** A correction was made 291-292.

- Line 334: I would suggest removing the comma in “the roundworm, Strongyloides stercoralis”

**Answer:** The sentence has been revised as

“This was followed by infection with the roundworm *Strongyloides stercoralis* which had a prevalence of 4.4%.” line 327-328.

- Grams abbreviation: I would recommend “g” instead of “gm”, unless the journal recommends otherwise (lines 390 and 409).

**Answer:** Correction was made in lines 383 and 401.

- Line 421: Change “mlL” for “mL”.

**Answer:** A correction was made.

- Line 422: I believe “<100 µg/mL” should read “100 ng/mL”

**Answer:** A correction was made in line 414.

- Line 425: For consistency, I recommend changing “epg” for “EPG”.

**Answer:** A correction was made.

- Line 469: “This is the Table 5 legend” should be removed.

**Answer:** “This is the Table 5 legend” has been removed.

- Line 478: Add % symbol to 91.6.

**Answer:** this is done by adding % in line 482.

- Line 479: I suggest changing “fourfold” to “four-fold”.

**Answer:** A correction was made in line 482.

- Line 519: Should “originated” read “originates”?

**Answer:** A correction was made in line 529. **Additional responses to Editor**

1) Please ensure that the CRediT author contributions listed for every co-author are completed accurately and in full.
At this stage, the following Authors/Authors require contributions: Phattharaphon Wongphutorn, Watchalin Loilome, Chaiwat Tawarungruang, Bandit Thinkhamrop, Chanika Worasith, Nisana Namwat, Chatanun Eamudomkarn, Simon D Taylor-Robinson, Attapol Titapun, Anchalee Techasen, Jiraporn Sithithaworn, Kulthida Y Kopolrat, Melissa R Haswell, Thomas Crellen, Samarn Futrakul, and Paiboon Sithithaworn. Please ensure that the full contributions of each author are acknowledged in the "Add/Edit/Remove Authors" section of our submission form.
The list of CRediT author contributions may be found here: <https://journals.plos.org/plosntds/s/authorship#loc-author-contributions>
**Answer :** Revised Author Contribution for each author was done in the Editorial Manager.

2) Thank you for stating"I have no competing interest."

Please include a completed 'Competing Interests' statement in the text box when submitting your production task, including any COIs declared by your co-authors, written in full sentences.
If you have no competing interests to declare, please state "The authors have declared that no competing interests exist". You may also provide an updated statement via email.
**Answer:** This is done by adding the text “The authors have declared that no competing interests exist" in the Editorial Manager
3) We do not publish any copyright or trademark symbols that usually accompany proprietary names, eg ©,  ®, or TM  (e.g. next to drug or reagent names). Therefore please remove all instances of trademark/copyright symbols throughout the text, including:  Parasep® on line no  684.
Answer: This is done.
4) Thank you for stating"All participants aged 18 years and over provided written informed consent, while parents or legal guardians provided consent for participants aged 15-17 years.

Please be so kind and specifically state whether written or verbal consent was obtained form the parents/legal guardians.

NOTE:  If child participants, the statement must declare that formal consent (must state whether verbal/written)was obtained from the parent/guardian.].
Answer: This is done.

5) We notice that your supplementary figures are uploaded with the file type 'Figure'. Please amend the file type to 'Supporting Information'. Please ensure that each Supporting Information file has a legend listed in the manuscript after the references list.

Answer: This is done.

6) We notice that your supplementary Figures are included in the manuscript file. Please remove them and upload them with the file type 'Supporting Information'. Please ensure that each Supporting Information file has a legend listed in the manuscript after the references list.

7) Some material included in your submission may be copyrighted. According to PLOSu2019s copyright policy, authors who use figures or other material (e.g., graphics, clipart, maps) from another author or copyright holder must demonstrate or obtain permission to publish this material under the Creative Commons Attribution 4.0 International (CC BY 4.0) License used by PLOS journals. Please closely review the details of PLOSu2019s copyright requirements here: PLOS Licenses and Copyright. If you need to request permissions from a copyright holder, you may use PLOS's Copyright Content Permission form.
Please respond directly to this email and provide any known details concerning your material's license terms and permissions required for reuse, even if you have not yet obtained copyright permissions or are unsure of your material's copyright compatibility. Once you have responded and addressed all other outstanding technical requirements, you may resubmit your manuscript within Editorial Manager. 
Potential Copyright Issues:

Thank you for stating" "Thematic maps were used to depict the spatial distribution of O. viverrini infection using Quantum GIS (<https://www.qgis.org/en/site/>)"."

i) Figure 2. Please (a) provide a direct link to the base layer of the map (i.e., the country or region border shape) and ensure this is also included in the figure legend; and (b) provide a link to the terms of use / license information for the base layer image or shapefile. We cannot publish proprietary or copyrighted maps (e.g. Google Maps, Mapquest) and the terms of use for your map base layer must be compatible with our CC BY 4.0 license.
Note: if you created the map in a software program like R or ArcGIS, please locate and indicate the source of the basemap shapefile onto which data has been plotted.
If your map was obtained from a copyrighted source please amend the figure so that the base map used is from an openly available source. Alternatively, please provide explicit written permission from the copyright holder granting you the right to publish the material under our CC BY 4.0 license.
If you are unsure whether you can use a map or not, please do reach out and we will be able to help you. The following websites are good examples of where you can source open access or public domain maps:
* U.S. Geological Survey (USGS) - All maps are in the public domain. ([http://www.usgs.gov](http://www.usgs.gov/))
* PlaniGlobe - All maps are published under a Creative Commons license so please cite u201cPlaniGlobe, [http://www.planiglobe.com](http://www.planiglobe.com/), CC BY 2.0u201d in the image credit after the caption. (<http://www.planiglobe.com/?lang=enl>)
* Natural Earth - All maps are public domain. (<http://www.naturalearthdata.com/about/terms-of-use/>).

**Answer :** The prevalence map was created in R software using shape files from UN-OCHA (https://data.humdata.org/dataset/geoboundaries-admin-boundaries-for-thailand) which are licensed under a Creative Commons Attribution 4.0 International license. The text is added in the material & method, line 294-297.

8) In the online submission form you indicate that your data is available from a co-author on this manuscript. According to our Data Policy, data must either be included with the submission, be placed in a public repository,
or available from a third party contact point who must not be an author on the manuscript.  Please revise your data statement to a non-author institutional point of contact, such as a data access or ethics committee,  and send this to us via return email. Please also include contact information for the third party organization  (this contact should be a central address and not a named person), and please include the full citation of where the  data can be found.

Answer : Supporting Information is updated as follow;

**Supporting information**

**S1 Text. Sample size calculation**

(DOCX)

**S1 Fig.** **Comparative prevalence rates of *O. viverrini* infection by FECT and urine antigen assays in 21 provinces of Thailand (n=18,062)**. Dark bars indicate prevalence as determined by FECT; superimposed white bars indicate prevalence measured using urinary antigen detection by ELISA. Bars are arranged in order of ascending prevalence based on FECT results.

(TIF)

**S2 Fig.** **Frequency distribution of *O. viverrini* antigen and egg in feces**. The fecal egg counts frequency (median = 16.9 EPG) (A), the urine antigen concentration (median = 47.8 ng/ml) among antigen-positive individuals (B) within the sample population in Thailand.

(TIF)

**S1 Checklist. STARD Checklist.**

(DOC)

**S1 Data files.**

(XLSX)

9) In the online submission form, you indicated that The data that support the findings of this study are available from the corresponding author upon reasonable request.. All PLOS journals now require all data underlying the findings described in their manuscript to be freely available to other researchers, either
1. In a public repository
2. Within the manuscript itself
3. Uploaded as supplementary information.
This policy applies to all data except where public deposition would breach compliance with the protocol approved by your research ethics board. If your data cannot be made publicly available for ethical or legal reasons (e.g., public availability would compromise patient privacy), please explain your reasons by return email and your exemption request will be escalated to the editor for approval. Your exemption request will be handled independently and will not hold up the peer review process, but will need to be resolved should your manuscript be accepted for publication. One of the Editorial team will then be in touch if there are any issues.
**Answer:** This is done. The S1 Data files is uploaded as supplementary information.

10) Please ensure that the funders and grant numbers match between the Financial Disclosure field and the Funding Information tab in your submission form. Note that the funders must be provided in the same order in both places as well.
Currently, order of grants are different in both places.
**Answer**: the order grants in the Financial Information and Financial Disclosure filed are revised as suggested. The funder URLs are also given.
