# Supplementary material for: Large-scale epidemiology of opisthorchiasis in 21 provinces in Thailand based on diagnosis by fecal egg examination and urine antigen assay and analysis of risk factors for infection
Source: PLoS Negl Trop Dis. 2025 Jul 16;19(7):e0013095. doi: 10.1371/journal.pntd.0013095 (PMC12313068; doi:10.1371/journal.pntd.0013095)
Supplement: Attachment — Submitted filename: RtoR_R2.docx [file pntd.0013095.s009.docx]

**Reviewers' comments:**

Reviewer's Responses to Questions

**Key Review Criteria Required for Acceptance?**

As you describe the new analyses required for acceptance, please consider the following:

**Methods:**

-Are the objectives of the study clearly articulated with a clear testable hypothesis stated?

-Is the study design appropriate to address the stated objectives?

-Is the population clearly described and appropriate for the hypothesis being tested?

-Is the sample size sufficient to ensure adequate power to address the hypothesis being tested?

-Were correct statistical analysis used to support conclusions?

-Are there concerns about ethical or regulatory requirements being met?

Reviewer #1: A revision was made as suggested. The way the authors use standalone FECT and composite results is acceptable.

Reviewer #2: No further comments regarding the Methods section.

**Results:**

-Does the analysis presented match the analysis plan?

-Are the results clearly and completely presented?

-Are the figures (Tables, Images) of sufficient quality for clarity?

Reviewer #1: Results are now better stratified (age intervals corrected to 10-year gaps). Analysis more clearly distinguishes the groups (FECT+/Urine+, FECT-/Urine-, etc.), but critical evaluation is limited when discordant results occur (FECT-/Urine+). The authors might need to state this limitation in the manuscript.

Answer : The limitation of urine assay regarding discordance with FECT is explained in more detail in 2 points in Discussion section in line 487-493 and line 563-566.

Reviewer #2: - Line 428 still shows 50.9% in positive rate for urine ELISA. Should this be 50.3%, same as the reported prevalence by urine ELISA?

Answer : For clarity we have revised the sentence and explained the subset samples used for diagnostic accuracy analysis and have removed the prevalence in question.

- Table 3: Please revise numbers in the 2x2, as not all of them add up to the total referred. The previous table had some diagnostic accuracy parameters (sensitivity, specificity, PPV, NPV...). Could the authors include (some of) them, as they were in the previous version, for each scenario, “a” and “b”?

Answer :Table 3 is now revised to include 2x2 data and diagnostic performance parameters for primary and composite reference standards as suggested.

**Conclusions:**

-Are the conclusions supported by the data presented?

-Are the limitations of analysis clearly described?

-Do the authors discuss how these data can be helpful to advance our understanding of the topic under study?

-Is public health relevance addressed?

Reviewer #1: Conclusions slightly softened, but the potential for overstatement of urine assay performance persists without robust justification. The authors might still need to state this limitation in the manuscript.

Answer : Please see the answer to reviewer #1 above.

Reviewer #2: No further comments regarding the Conclusions section.

**Editorial and Data Presentation Modifications?**

Use this section for editorial suggestions as well as relatively minor modifications of existing data that would enhance clarity. If the only modifications needed are minor and/or editorial, you may wish to recommend “Minor Revision” or “Accept”.

Reviewer #1: (No Response)

Reviewer #2: (No Response)

**Summary and General Comments:**

Use this section to provide overall comments, discuss strengths/weaknesses of the study, novelty, significance, general execution and scholarship. You may also include additional comments for the author, including concerns about dual publication, research ethics, or publication ethics. If requesting major revision, please articulate the new experiments that are needed.

Reviewer #1: The work remains highly novel (the first large-scale urine antigen epidemiological survey across Thailand) and impactful.

Reviewer #2: (No Response)

PLOS authors have the option to publish the peer review history of their article ([what does this mean?](https://track.editorialmanager.com/CL0/https:%2F%2Fjournals.plos.org%2Fplosntds%2Fs%2Feditorial-and-peer-review-process%23loc-peer-review-history/1/010f01964472e99d-8fb8a2a7-f0f1-4f9a-8e5d-6ea02b193a0d-000000/Np0TdNRDR_mO5b560lKQcH4U0RFWt7urJAz6XoFyTZ8=207)). If published, this will include your full peer review and any attached files.

If you choose “no”, your identity will remain anonymous but your review may still be made public.

**Do you want your identity to be public for this peer review?** For information about this choice, including consent withdrawal, please see our [Privacy Policy](https://track.editorialmanager.com/CL0/https:%2F%2Fwww.plos.org%2Fprivacy-policy/1/010f01964472e99d-8fb8a2a7-f0f1-4f9a-8e5d-6ea02b193a0d-000000/-OGHYk1YKpIxc-GNoqLeBSqxfRfdFRGB59bchscvNCo=207).

Reviewer #1: No

Reviewer #2: **Yes:** Elías Kabbas-Piñango
